# Genetic and Epigenetic Mechanisms Linking Air Pollution and Congenital Heart Disease

**DOI:** 10.3390/jcdd3040032

**Published:** 2016-11-29

**Authors:** Cecilia Vecoli, Silvia Pulignani, Maria Grazia Andreassi

**Affiliations:** Institute of Clinical Physiology-National Research Council (CNR), Via Moruzzi, 1 56124 Pisa, Italy; pulignani@ifc.cnr.it (S.P.); andreas@ifc.cnr.it (M.G.A.)

**Keywords:** congenital heart disease, air pollutions, individual susceptibility, epigenetics

## Abstract

Epidemiological studies strongly suggest that parental air pollutants exposure during the periconceptional period may play a major role in causing fetal/newborn malformations, including a frequent heterogeneity in the methods applied and a difficulty in estimating the clear effect of environmental toxicants. Moreover, only some couples exposed to toxicants during the pre-conception period give birth to a child with congenital anomalies. The reasons for such phenomena remain elusive but they can be explained by the individual, innate ability to metabolize these contaminants that eventually defines the ultimate dose of a biological active toxicant. In this paper, we reviewed the major evidence regarding the role of parental air pollutant exposure on congenital heart disease (CHD) risk as well as the modulating effect on detoxification systems. Finally, major epigenetic alterations induced by adverse environment contaminants have been revised as possible mechanisms altering a correct heart morphogenesis.

## 1. Introduction

Air pollution is a universal issue and a major public health concern since it can affect everyone and can cause numerous heterogeneous adverse health events including asthma attacks, cancer and cardiovascular diseases [1,2,3]. 

Environmental noxae have effects on all age groups but fetuses are definitely the most vulnerable. Thus, fetal environment is critical for correct development because fetal sensitivity and the ability to absorb toxins and environmental chemical pollutants are higher than in adults [4,5]. 

Accordingly, epidemiological evidence estimates an increased number of congenital malformations in high-impact areas, especially congenital heart diseases (CHDs) [6]. CHD is the most common congenital abnormality and one of the leading causes of newborn death in developed and developing countries. The European Surveillance of Congenital Anomalies (EUROCAT) estimated that about 23% of all congenital anomalies concerns heart defects [7]. Anyhow, the precise etiological basis underlying the majority of CHD cases remains elusive. 

Genetic factors have been recognized for a long time as the predominant cause of CHDs. Anyhow, the majority of cases is sporadic and without a familial history of disease, and the known genetic causes (such as chromosomal anomalies, Mendelian syndromes, and non-syndromal single-gene disorders) are estimated to account for less than 20% of cardiac malformations [8]. The other 80% has an unknown etiology that follows a multifactorial inheritance model, in which genetic factors are implicated and where environmental factors contribute heavily [9]. From this prospective, clear information on the impact of the environment is fundamental, firstly because the fraction of cases with CHD attributable to identifiable and potentially modifiable factors might be as high as 30% [10]. A better knowledge of these changeable risks might represent a real opportunity for prevention of potential disease.

Actually, although substantial data have demonstrated that the exposure to toxicants during the periconceptional period increases the risk of CHD [11,12,13], only some couples exposed to toxicants give birth to a child with CHD. The reason for this remains elusive, but it might be associated with the innate capability to metabolize noxious compounds.

**Figure 1:**
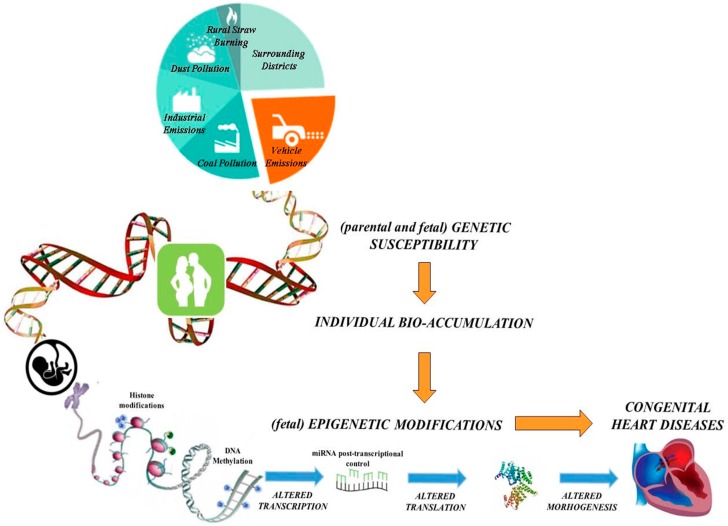
Genetic susceptibility and epigenetic mechanisms linking air pollution and congenital heart disease.

Additionally, there is compelling evidence that periconceptional exposure to environmental xenobiotics adversely affects fetal development through alterations in epigenetic mechanisms [14], which produce heritable modifications in gene expression without changes in DNA sequence. In fact, abnormal fetal epigenetic rearrangement that is strictly dependent on environmental stressors might predispose certain individuals to different diseases, including CHD [15]. Our proposed mechanisms linking air pollution and congenital heart disease are schematized in Figure 1. Briefly, we hypothesize that parental and/or fetal genetic background (genetic susceptibility) may predispose individuals to a different degree of CHD risk associated with air pollutant exposure (mainly due to industrial emissions, urbanization and transport of goods and people by fuel-engine motor vehicles). Genetic variants can modulate the activity of metabolizing enzymes contributing to the effective amount of contaminant(s) in the body (individual bio-accumulation). The toxic compounds accumulated may modify the fetal epigenetic profile, increasing the possibility to develop abnormal heart morphogenesis.

## 2. Air Pollutant Exposure and CHD Risk: Epidemiological Evidences

The majority of evidence suggests that maternal exposure to harmful compounds is a risk factor increasing the incidence of CHD, although some findings fail to confirm this. Conversely, only few studies have been performed to evaluate the impact of male periconceptional exposure on offspring's health. Indeed, the risk of toxicant exposure in the male reproductive field has long been focused on its impact on male (in)fertility (rather than on offspring’s health). However, the studies available suggest a key role also for the paternal exposure in CHD risk of progeny [16,17,18,19]. In the past decade, the number of studies investigating the association between CHD and air pollution has increased but the results remain inconsistent and controversial (Table 1) [20,21,22,23,24,25,26,27,28,29,30,31,32].

Environmental pollutants of concern regarding health impact include a wide range of airborne contaminants. The major air pollutants due to industrial emissions, urbanization and transport of goods and people by fuel-engine motor vehicles are carbon monoxide (CO), nitrogen oxides (NO_x_)—especially nitrogen dioxide (NO_2_)—sulfur dioxide (SO_2_), and particulate matter (PM). 

Carbon monoxide, in addition to being one of the main air pollutants, is the major constituent of tobacco smoke. CO dissolved in maternal plasma crosses the placental barrier and is detectable in fetal circulation in the form of carboxyhemoglobin [33]. Fetal tissues are more at risk of hypoxia due to maternal exposure to CO compared to maternal tissues [34,35]. It has been suggested that even at non-toxic levels of CO for mothers, CO fetotoxicity may occur [34,36]. In 2011, Dadvand et al. [20] performed a case control study investigating the association between maternal exposure to ambient pollutants (PM_10_, SO_2_, NO, O_3_, and CO) and the occurrence of CHD in the population of Northeast England (1993–2003). Specifically regarding CO, maternal exposure was associated with the occurrence of specific congenital malformations of cardiac septa (OR = 2.330; 95%CI 1.748–3.102), ventricular septal defect (OR = 2.634; 95%CI 1.871–3.707), and congenital pulmonary valve stenosis (OR = 2.682; 95%CI 1.298–5.534). The results obtained by Dadvand et al. [20] were consistent with previous reports including Ritz et al. [22] , who reported an association between CO and isolated ventricular septal defects (OR = 1.62 for 2nd quartile; OR= 2.09 for 3rd quartile, and OR = 2.95 for 4th quartile) but were not supported by others [21,23,24,25]. 

NO_x_ is an irritant gas, produced from the reaction of nitrogen and oxygen gases in the air during combustion. A statistically significant positive association with both spatial and spatiotemporal exposure to traffic-related air pollution in Barcelona (Spain) has been estimated for NO_2_/NO_x_ and coarctation of the aorta (OR_spatiotemporal_ = 1.15; 95%CI 1.01–1.31) and digestive system anomalies (OR_spatiotemporal_ = 1.11; 95%CI 1.00–1.23) [25]. These results were consistent with other findings [26,32] but conflicted with other ones [27,28]. Interestingly, a recent meta-analysis conducted to assess the association between air pollution and the risk of congenital anomalies found that only NO_2_ concentration was significantly associated with coarctation of the aorta (OR = 1.20 per 10 ppb; 95%CI 1.02–1.41) [37]. 

SO_2_ is recognized as a toxic air pollutant although little information is available regarding the mechanism by which it can disturb the normal development of the embryo or fetus. It potentially involves oxidative damage caused by free radicals formed during sulfite oxidation. Recently, in a population-based case control study, Gianicolo et al. [38] found an increased prevalence of CHD among live births in Brindisi (Italy)—area at high environmental impact—also in comparison with the pool of EUROCAT registries [38]. Specifically, the increased risk of CHD (OR = 3.21; 95%CI 1.42–7.25, p-trend = 0.01)—especially ventricular septal defects (OR = 4.57; 95%CI 1.31–15.96, p-trend = 0.048)—was associated with the exposure of the highest daily average values of SO_2_ [29]. The results obtained by Gianicolo et al. were consistent with those found by others including Gilboa et al. [21] and Dolk et al. [30], especially regarding the increased risk of ventricular septal defects, atrial septal defects and tetralogy of Fallot. Conversely, they differed from those obtained by Dadvand et al. [32], which showed an inverse relationship between maternal exposure to SO_2_ and risk of ventricular septal defect similarly to Strickland et al. [23] and Hansen et al. [24]. More recently, the association between the exposure to ambient SO_2_ during pregnancy and an increase in birth risk defects has been confirmed in a retrospective study conducted in Anqing city of Eastern China [39].

Conflicting results emerge also in the association analyses between PM and congenital cardiac anomalies. Stingone et al. [26] in a study involving the mothers of National Birth Defects Prevention Study (a nine-state case-control study) found that exposure to fine particulate matter in weeks 2–8 of pregnancy was positively associated with hypoplastic left heart syndrome (90th/10th centile contrast: OR = 2.04; 95%CI 1.07–3.89) but inversely associated with atrial septal defects (OR for 50th–90th/10th contrast = 0.50; 95%CI 0.38–0.65; OR for 90th/10th contrast = 0.54; 95%CI 0.35–0.81) by using the hierarchical regression models of the 7-week average exposure to individual PM and CHDs [26]. In a study of residents of San Joaquin Valley, California, investigators showed that higher levels of maternal exposure to PM_10_ were associated with pulmonary valve stenosis (OR for 4th quartile = 2.6; 95%CI 1.2–5.7) and perimembranous ventricular septal defects (OR for 3rd quartile = 2.1; 95%CI 1.1–3.9) after adjusting for maternal race/ethnicity, education and multivitamin use. PM_2.5_ was associated with transposition of the great arteries (OR for 3rd quartile = 2.6; 95%CI 1.1–6.5) but inversely associated with perimembranous ventricular septal defects (OR 4th quartile = 0.5; 95%CI 0.2–0.9) and secundum atrial septal defects (OR 4th quartile = 0.5; 95%CI 0.3–0.8) [27]. Likewise, more recently, Schembari et al. [25] described an inverse association between PM_2.5_ and ventricular septal defect (OR = 0.83; 95%CI 0.72–0.97) by estimating exposure during the weeks 3–8 of pregnancy in a population of 106 cases and 903 controls within the years 2000–2006 in the Barcelona area [25]. No other significant associations between maternal exposure to PM_2.5_ and risk of congenital heart defects or neural tube defects were observed. In a study performed in the Tel-Aviv region (Israel), an inverse association between patent ductus arteriosus cases and exposure to PM_2.5_ (OR = 0.78; 95%CI 0.68–0.91) as well as a positive association between PM_10_ and multiple congenital heart defects was observed (OR = 0.05; 95%CI 1.01–1.10) [28] according to previous works [21,27,30,31]. Other studies found no associations [20,24,25,26].

All the contradictory studies on the potential impact of environmental exposures in affecting congenital anomalies have been recently reviewed in two different meta-analyses [37,40]. The two studies found some evidence for an effect of ambient air pollutants on congenital cardiac anomaly risk although the most recent study showed only a significant association between NO_2_ concentrations and aortic coarctation [37]. All the authors suggested the need to improve the exposure assessment, to harmonize the outcomes as well as a better control of confounders in future congenital anomalies research in this area. Indeed, different explanations are plausible to explain the inconsistencies that remain from epidemiological studies. First of all, the different criteria used for definition and classification of congenital cardiac anomaly subgroups. In general, congenital heart defects form a very heterogeneous set of conditions, notoriously difficult to classify [24,41]. Several classification systems have been proposed [41,42], but diagnostics information in routine registries may often not be specific enough to apply these. Ventricular septal defects, for example, were treated as one anomaly group by some studies, as four different anomalies by another one, and were excluded in yet another [20,21,22,23,24,30,32]. 

Exposure assessment is also challenging since the air pollution monitoring is dependent on the contributing sources and can vary strongly by geographical area over a relatively short distance and temporal patterns. “Averaging” of exposure might mask the identification of some who have been unexposed and others who have been highly exposed, as is the case of population living upwind or downwind of pollution sources or living closest to them on windless days. 

Moreover, the presence of multiple confounding factors (parental smoking, occupation and age as well as season conception) [22,36] makes difficult a real measure, causing underestimation of the true association between air pollution and the occurrence of CHD. Additionally, exposure estimation as well as risk factors are self-reported and extrapolated from the responses of a questionnaire. Thus, random misclassification of exposure and recall biases cannot be excluded. Lack of data on individual direct levels of exposure, small sample size, and other lifestyle factors may represent confounding elements in the area of residence [43]. Finally, epidemiological studies show a total lack of interaction between exposure to toxicant and individual genetic susceptibility, which could explain why only some couples exposed to air contaminants (during the periconceptional period) give birth to a child with congenital heart anomalies [44].

## 3. Maternal Susceptibility to Air Pollution and CHD Risk

The dangerous effects of air contaminants in the human body ultimately depends on the effective amount of contaminant(s) in the body that, in turn, strictly depend on the individual capability to activate and detoxify the toxicant(s).

Accordingly, evidence suggests a strict interaction between environmental exposure and maternal genetic capability to metabolize toxins in increasing the risk of several congenital diseases [45,46]. In fact, the human body, in order to protect itself against the potential harmful insults, is equipped with xenobiotic metabolizing enzymes that are able to biotransform and metabolize xenobiotics or foreign compounds, including different kinds of pollutants. Xenobiotic metabolizing enzymes include a variety of enzymes such as cytochrome P450 (P450 or CYP), epoxide hydrolase, glutathione transferase, sulfotransferase, NAD[P]H quinone oxidoreductase 1, and aldo-keto reductase. These enzymes mainly participate in the conversion of xenobiotics to more polar and water-soluble metabolites, which are readily excreted from the body. Thus, the so-called “phase I” enzymes are mainly involved in the activation of chemical toxicants by introducing reactive or polar groups into xenobiotics. For instance, the superfamily of cytochrome P450 enzymes catalyzes oxidation of a large number of endogenous (e.g., hormones and fatty acids) and exogenous (e.g., polycyclic aromatic hydrocarbons (PAH), aromatic amines and mycotoxins) chemicals. Then, the “phase II” enzymes (e.g., epoxide hydrolase, glutathione S-transferases (GST), sulfotransferases) are involved in the detoxification of the modified compounds by conjugating them with glutathione, glucuronide or sulfate to produce products quickly excreted. 

Genetic variants able to affect the activity of xenobiotic metabolizing enzymes make each person differentially able to metabolize these compounds. In other words, each person has a specific sensitivity to adverse effects of environmental toxicants.

Thus, the genetic background interacting with environmental exposure contributes to the inter-individual variations of genetic susceptibility to different disease including cancer [47] or autism [48,49]. Several studies have evidenced the major role of specific polymorphic variants in toxicant’s detoxification genes in association with congenital malformations including heart abnormalities [50,51,52]. For instance, Wang et al. showed that the C3435T polymorphism in the ATP-binding cassette (ABC) transporters 1 (ABCB1) gene increases the risks of CHD, particularly for septal defects, in a Han Chinese population when the mothers are exposed to noxious compounds during the periconceptional period [53].

There is increasing evidence that the placenta expresses a range of transporters capable of controlling the transplacental disposition of many toxicant agents [54,55]. ABCB transporters are able to efflux environmental toxicants or drugs ingested by the mother into the maternal circulation. In particular, the expression of transporter proteins at placenta levels is important in protecting the fetus from toxic xenobiotics. The *ABCB1* or *MDR1* gene in humans is the best characterized for drug efflux transporters [56]. Functional single-nucleotide polymorphisms (SNPs) in *ABCB1* genes may influence expression and activity of this protein in the placenta, leading to altered fetal exposure to xenobiotics, and subsequent increase in the risk of complex genetic disorders or birth defects. 

Interestingly, we recently showed that exposure to toxicants for both parents affects the risk of children with CHD, supporting the hypothesis of the pivotal influence of the environmental risk factors for congenital malformations [18]. In addition, a gene-environment analysis suggested that null GSTs genes could modify a person’s risk of toxicant exposure-induced disease. Specifically, children with the combined GSTM1 and GSTT1 null genotypes had a greater risk of having CHD than children carrying wild-type GST genes when both parents were exposed [18].

Previous studies have already demonstrated that the deletion in the GSTM1 and GSTT1 genes contributed to the development of other congenital malformations, such as oral cleft defects [52]. In particular, an elevated relative risk of cleft palate in infants with the GSTT1 null genotype, whose mothers were exposed to occupational chemicals, has been detected [57].

Recently, developmental interference with endogenous Aryl Hydrocarbon Receptor (AHR) functions has been shown to adversely affect the cardiovascular system in various experimental models, and have implicated the AHR in the etiology of cardiovascular disease. Genome-wide studies in mouse embryonic stem cells have shown that disruption of endogenous AHR expression perturbs cardiomyocyte differentiation, underscoring a critical role for the receptor in a complex regulatory target network for cardiogenesis and cardiovascular homeostasis [58]. The effect of *in utero* AHR disruption during early life embryonic days delineated covert cardiac morphological functional effects, accompanied by many dysregulated signaling pathways involved in cardiogenesis, cardiac function, and mitochondrial function [59]. Recently, Carreira et al. confirmed the central role of the AHR signaling network in cardiovascular function and dysfunction, making it an important gene environment nexus in environmental cardiac injury. To date, specific studies that have aimed to investigate the role of specific metabolizing genes and air pollutants in relationship with CHD risk are missing [60] but all of the above-mentioned findings call for further research to determine the interactions between the individual genetic makeup and environmental factors.

## 4. Epigenetics: A Possible Link between Air Pollution and Congenital Heart Disease

Air pollutants can affect the molecule of DNA, inducing both changes in the DNA sequence (including nucleotide mutations and chromosomal aberrations) and epigenetic alterations that may mediate the toxicity of several environmental pollutants [61]. Epigenetics is defined as heritable changes in gene activity and expression that occur without alteration in the DNA sequence. 

Epigenetic profile could make individuals differentially vulnerable to environmental insults or to genetic variants. Epigenetic modifications can arise during both cell differentiation and embryonic morphogenesis, or during the mitotic divisions of a cell and play a critical role in the regulation of different genomic functions. 

The epigenome is susceptible to (dys)regulation throughout life but, during embryogenesis it is supposed to be very vulnerable to external factors because of the rapid division and epigenetic remodeling of cells [62,63]. 

It is known that environmental chemical exposures *in utero* can influence the epigenome, resulting in congenital defects or diseases developed later in life [64]. Conversely, abnormal epigenetic profiles in sperm may result in genomic instability, impaired fertility and compromised spermatogenesis. Furthermore, a high incidence of diseases has been recorded in the offspring of men exhibiting spermatic DNA damage [65]. Interestingly, epigenetics effects can be life-long and may even be transferred to next generation.

The two more studied kinds of epigenetic information that can be inherited with chromosomes are DNA methylation and changes in chromatin proteins, usually due to modifications in histone tails. Methylation was the first epigenetic mechanism to be discovered. In mammals, DNA methylation is essential during embryogenesis when changes dynamically adapt embryos to make them fit for further differentiation [66,67]. Recently, Janssen et al. (2013) observed a lower degree of placental global DNA methylation in association with exposure to particulate air pollution in early pregnancy, including the critical stages of implantation [68]. Placental tissue has emerged as a popular candidate for analysis of DNA methylation. Appropriate placental gene expression is paramount to fetal regulation during pregnancy, and alterations from this have been linked to other pathologies, including intrauterine growth restriction and trophoblastic disease [69,70]. The strong association between gene expression and DNA methylation point towards a potentially significant mechanism of prenatal programming. These modifications might provide a plausible link between particulate air pollution and alterations in gene expression that might lead to disease phenotypes related to fetal programming. Green and Marsit (2015) recently reviewed the relevant literature relating DNA methylation in multiple tissues at or near delivery to several prenatal environmental toxicants and stressors [71]. The authors concluded that, all together, the studies suggested an important role of DNA methylation in mediating the effects of the intrauterine environment on children’s health. 

Other authors have examined the role of environmental factors on DNA methylation levels. Baccarelli and colleagues (2009) showed that blood DNA methylation in the LINE-1 repetitive element was decreased in elderly individuals of the Normative Aging Study with recent exposure to higher levels of traffic particles [72]. Another study within the same elderly cohort found that prolonged exposure to black carbon and sulfate particles is associated with hypomethylation of Alu and LINE-1 in leukocytes, respectively [73]. Finally, several studies also report associations of gene-specific DNA methylation in leukocytes and exposure to airborne polycyclic aromatic hydrocarbons and PM [74,75]. In contrast to particulate exposure, arsenic was positively associated with DNA methylation in LINE-1 repeated element in both maternal and fetal leukocytes [76].

The aberrant methylation status of cardiac transcription factors, *NKX2-5* and *HAND1*, was reported to be negatively correlated with their corresponding mRNA expression in patients with tetralogy of Fallot [77]. Recently, Serra-Juhé et al. (2015) showed that some epigenetic alterations were present in the DNA of developing heart tissue of fetuses with CHD, both isolated and syndromic [78]. These methylation aberrations might contribute to the etiology and/or pathogenesis of the malformation through deregulation of gene expression during heart development [78].

Chromatin remodeling and histone modification have substantial roles in activating or silencing gene expression. Histones are globular proteins that undergo posttranslational modifications that alter their interaction with DNA and other nuclear proteins [79,80]. H3 and H4 histones have long tails protruding from the nucleosome, which can be covalently modified by several mechanisms including acetylation, methylation, and ubiquitination that influence chromatin structure and gene expression. The function of transcription factors is intimately associated with the status of the chromatin at particular targets. Interactions between chromatin remodeling factors and transcription factors provide an additional level of gene expression regulation. This regulation is crucial for normal heart development [81]. From a molecular point of view, histone modification is considered a central epigenetic mark like methylation, since chromatin structure plays a major role in the regulation of the genome. Histone deacetylases [HDACs] are a class of enzyme that, by removing acetyl groups from histone tails, compact the chromatin and repress the transcription [82]. Interestingly, an experimental animal study showed that HDAC1 and HDAC 2 play a role in cardiac morphogenesis, growth, and contractility. Indeed, the hearts of mutant mice displayed unusual morphological abnormalities of the right ventricular chamber [82]. Few studies are available concerning the association between air pollution and histone modification. Anyhow, there is evidence that PM_10_ exposure promotes inflammatory cytokine release in lung epithelial A549 cells by increasing histone acetyltransferases activity and acetylation of histone H4 [83]. Moreover, exposure to diesel exhaust particles has been shown to decrease the HDAC2 activity to increase the acetylation of histone H4 [84]. 

A third, more novel epigenetic mechanism is represented by microRNAs (miRNAs). MiRNAs are single-stranded RNAs of ≈21–23 nucleotides in length that are transcribed from DNA but not translated into proteins (non-coding RNAs). Mature miRNAs are partially complementary to one or more messenger RNA (mRNA) molecules. The main function of miRNAs is to down-regulate gene expression by interfering with mRNA functions [85,86]. MiRNA changes may be sensitive indicators of the effects of acute and chronic environmental exposure. Therefore, miRNAs are valuable novel biomarkers of exposure [87].

In healthy adults, the exposure to ambient particles altered the expression of candidate microRNAs in blood-leukocytes [88,89]. Inhalation of ozone was shown to disrupt miRNA expression profiles in human induced-sputum samples, and network analysis of 10 miRNAs with significantly increased expression levels revealed an association with diverse biological processes, including inflammatory and immune response signaling [90]. Interestingly, exposure to environmental agents induces altered miRNA expression patterns both in placental cell lines [91] and cord blood [92] which could potentially contribute to adverse fetal development and health outcomes later in life. A number of miRNAs has recently been identified as critical in the heart homeostasis [93]. For example, miR-1, the first one identified to play a key role in cardiomyocytes differentiation, is specifically expressed in cardiac precursor cells that specifically target regulators of muscle differentiation [94,95]. During cardiogenesis, an excess of miR-1 decreased the number of proliferating ventricular cardiomyocytes modulating regulatory proteins involved in the differentiation/proliferation balance control. In addition, overexpression of miR-1 decreased the level of Hand2 protein without changing its mRNA level, suggesting that Hand2 is a target of miR-1 during heart development [94]. Deletion of miR-1-2 results in heart defects that include ventricular septal defects leading to modifications in conduction system and in cardiomyocytes proliferation [96]. Our group found that functional genetic variants in the 3′UTR of transcription factor GATA4 might contribute to the pathogenesis of CHDs, likely by affecting the miRNA post-transcriptional control [97]. 

Finally, Zhu et al. [98] recently hypothesized that miRNAs in maternal serum could act as candidate biomarkers for the prenatal detection of fetal CHD in early pregnancy. This group identified four significantly up-regulated miRNAs in mothers carrying fetuses with CHD. Specifically, the combination of these four differentially expressed miRNAs could act as novel non-invasive biomarkers for the prenatal detection of fetal CHD. This idea is in its infancy and there are certainly some limitations, i.e., sample size, huge heterogeneity of CHD and conceivably variability within the mother populations themselves, but it is a promising clinical application for the diagnosis of fetal CHD. 

Further research is required to accurately explore the possibility that epigenetic alterations—highly changeable under different environmental stressors—can be used in clinical practice for prenatal detection in CHD. In particular, focused studies are needed in order to investigate the impact of air pollution on epigenetic alterations associated with CHD. Understanding how epigenetic alterations are associated with abnormal heart development could be useful in order to finalize preventive strategies.

## 5. Conclusions

Altogether, the data available so far suggest a possible role of air pollution in the onset of congenital heart malformations. New focused studies should be designed in order to confirm this hypothesis. Indeed, only a precise (direct) measure of exposure level together with the knowledge of individual susceptibility to contaminants—as well as the identification of genetic factors able to define the predisposition to disease—could help to explain the real effects of air contaminants and make possible effective prevention policies and interventions. Moreover, future prospective investigations need to determine whether exposed subjects develop epigenetic alterations over time and, in turn, whether these alterations that regulate the expression of genes involved in heart development increase the risk of CHD in their offspring. Information about molecular mechanisms involved in the onset of heart abnormalities is fundamental in order to develop new strategies directed specifically at reducing the public health impact of CHD.

## Figures and Tables

**Table 1:** Overview of major epidemiological studies analyzing the impact of air pollution on congenital heart disease (CHD).

Reference	Setting	Study Design	Air Pollutants Measured	Air Pollutants Found Associated with CHD
Dadvand et al. 2011 [20]	Northeast of UK 1993–2003	Case-control, frequency matching	SO_2_, NO_2_, CO, PM_10_	NO_2_, CO
Gilboa et al. 2005 [21]	Texas, USA 1997–2000	Case-control, frequency matching	SO_2_, NO_2_, CO, PM_10_	SO_2_, CO, PM_10_
Ritz et al. 2002 [22]	California, USA 1987–1993	Case-control, no matching	CO	CO
Strickland et al. 2009 [23]	Atlanta, USA 1986–2003	Cohort	SO_2_, NO_2_, CO, PM_10,_ PM_2.5_	PM_2.5,_ NO_2_
Hansen et al. 2009 [24]	Brisbane, Australia 1998–2004	Case-control, individual matching	SO_2_, NO, CO, PM_10_	*No significant association*
Schembari et al. 2013 [25]	Barcelona, Spain 1994–2006	Case-control, no matching	NO_2_, NOx, PM_10_, PM_2.5_, PM_coarse_	NO_2_, PM_coarse_
Stingone et al. 2014 [26]	Nine U.S.states 1997–2006	Case-control, no matching	NO_2_, SO_2_, PM_10_, PM_2.5_, CO	NO_2_, PM_2.5_,
Padula et al. 2013 [27]	California, USA 1997–2006	Case-control, no matching	NO, NO_2_, PM_10_, PM_2.5_, CO	PM_10_, PM_2.5_
Agay-Shay et al. 2013 [28]	Tel-Aviv, Israel 2000–2006	Case-control, no matching	NO_2_, SO_2_, PM_10_, PM_2.5_, CO	PM_10_
Gianicolo et al. 2014 [29]	Brindisi, Italy 2000–2010	Case-control, individual matching	SO_2_	SO_2_
Dolk et al. 2010 [30]	Wessex, North West Thamas, Oxford and Northern of UK 1991–1999	Cohort	SO_2_, NO_2_, PM_10_	SO_2_, PM_10_
Kim et al. 2007 [31]	Seoul, Korea 2001–2004	Birth cohort	PM_10_	PM_10_
Dadvand et al. 2011 [32]	Northeast of UK 1985–1996	Case-control, frequency matching	SO_2_	*No significant association*
